# Characteristics of Bone-Conduction Devices Simulated in a Finite-Element Model of a Whole Human Head

**DOI:** 10.1177/2331216519836053

**Published:** 2019-03-17

**Authors:** You Chang, Stefan Stenfelt

**Affiliations:** 1 Department of Clinical and Experimental Medicine, Linköping University, Sweden

**Keywords:** bone conduction, bone-conducted sound, finite-element model, bone-conduction devices, human head

## Abstract

Nowadays, many different kinds of bone-conduction devices (BCDs) are available for hearing rehabilitation. Most studies of these devices fail to compare the different types of BCDs under the same conditions. Moreover, most results are between two BCDs in the same subject, or two BCDs in different subjects failing to provide an overview of the results between several of the BCDs. Another issue is that some BCDs require surgical procedures that prevent comparison of the BCDs in the same persons. In this study, four types of skin-drive BCDs, three direct-drive BCDs, and one oral device were evaluated in a finite-element model of the human head that was able to simulate all BCDs under the same conditions. The evaluation was conducted using both a dynamic force as input and an electric voltage to a model of a BCD vibrator unit. The results showed that the direct-drive BCDs and the oral device gave vibration responses within 10 dB at the cochlea. The skin-drive BCDs had similar or even better cochlear vibration responses than the direct-drive BCDs at low frequencies, but the direct-drive BCDs gave up to 30 dB higher cochlear vibration responses at high frequencies. The study also investigated the mechanical point impedance at the interface between the BCD and the head, providing information that explains some of the differences seen in the results. For example, when the skin-drive BCD attachment area becomes too small, the transducer cannot provide an output force similar to the devices with larger attachment surfaces.

## Introduction

Bone-conduction (BC) hearing is known as the perception of sound transmitted through the skull bone (Stenfelt, 2011; Stenfelt & Goode, 2005a). In BC sound transmission, sound is converted to vibrations that are transmitted through the skull bone directly to the cochlea. Consequently, BC sound can be audible without the interaction of the outer and middle ear. As a result, hearing devices based on BC sound transmission were designed to bypass the outer and middle ear. Nowadays, BC devices (BCDs) are widely used in many applications, such as communication systems, language development approaches, mitigation of stuttering, audiometric investigations, and hearing rehabilitation (Reinfeldt, Håkansson, Taghavi, & Eeg-Olofsson, 2015).

Increasing numbers of BCDs are available for communications, hearing rehabilitation, and hearing testing. Each BCD has a unique design with different geometries and masses; they connect to the skull using unequal methods (attached to the skin, anchored to the skull bone, or implanted in the skull bone) and are located at different positions on the head. According to the interface of the BC transducer and the skull, BCDs can be categorized as skin-drive BCDs, where the transducer is attached to the skin or direct-drive BCDs where the transducer is rigidly coupled to the skull bone (Reinfeldt, Håkansson, Taghavi, & Eeg-Olofsson, 2015).

Most of the BCDs are attached to the skin, or implanted into the skull bone, at the mastoid behind the ear canal opening or slightly further back. For example, currently, the most common type of BCD, the bone-anchored hearing aid (Baha®), is attached to a titanium implant at approximately 55 mm behind the ear canal opening in line with the upper part of the pinna (sometimes referred to as the Baha® position). In addition, there are BCDs, here termed oral devices, where the BCDs stimulate the ear by transmitting the BC vibration via a tooth to which the transducer is attached.

The characteristics of different BCDs have been discussed in the existing literature (Barbara, Perotti, Gioia, Volpini, & Monini, 2013; Reinfeldt, Håkansson, Taghavi, & Eeg-Olofsson, 2015; Syms & Hernandez, 2014; Wimmer et al., 2015). Some studies compared different BCDs (Hol, Nelissen, Agterberg, Cremers, & Snik, 2013; Stenfelt & Håkansson, 1999) but only regarding nonimplantable BCDs to one implantable BCD or a group of people using one type of implantable BCD to another group of people using another type of implantable BCD. The comparison between different BCDs, especially different implantable BCDs, in the same participant is uncommon. Due to individual differences to skin and skull bone thickness, skull geometry, mass, and composition, the responses of similar BCDs could differ between subjects. Moreover, due to the destruction of the skull bone during the implant surgery, it is almost impossible to compare different implantable BCDs in one individual.

One way to circumvent this problem is to evaluate the BCDs in a finite-element (FE) model of the head. Recently, a novel three-dimensional (3D) FE model of the human head, the LiUHead, was devised by Chang, Kim, and Stenfelt (2016) to be used for simulations of BC sound (Chang, Kim, & Stenfelt, 2018). The model is based on cryosectional images of an adult female (Visible Human Project^©^, http://vhnet.nlm.nih.gov/). The LiUHead was validated by comparing and correlating the simulation results with experimental data obtained from cadaver heads and living humans (Chang et al., 2016).

This BC model offers a unique opportunity to investigate BC sound transmission with different types of BCDs. These devices have often been evaluated by testing them in groups of people and reporting thresholds and speech perception abilities. Such evaluations are important for investigating functions of the entire systems but do not reveal the details and differences in BC sound transmission and the influences of the specific attachments between the device and the head. In this study, four types of skin-drive BCDs, three direct-drive BCDs, and one oral device with two stimulation positions were evaluated in terms of BC sound transmission from the interface between the BCD and skull and the inner ears. The aim of this study is to compare the BC characteristics of different BCDs and reveal the influence of the position and attachment method on the BC excitation of the cochlear promontory.

## Materials and Methods

### FE Model

**Figure 1.**
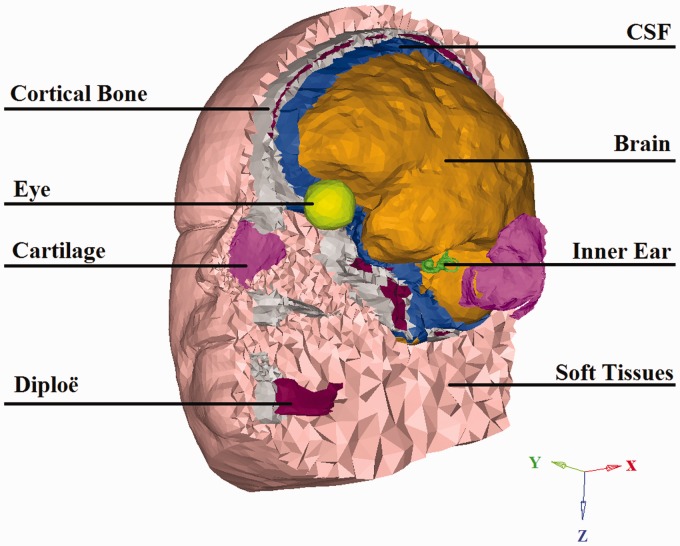
An illustration of the FE model LiUHead with its components. Details of the model and its components are described in the text. CSF = cerebrospinal fluid.

The LiUHead, which is an FE model of the whole head (Chang et al., 2016), is used for the simulations. The original FE model comprises 87,000 nodes and 481,000 four-nodded tetrahedron elements and includes eight domains: (a) the brain, (b) cerebrospinal fluid, (c) eye balls, (d) inner ears, (e) cartilages, (f) cortical bone (including teeth), (g) soft bone (diploë), and (h) soft tissues (Figure 1). The parameter values of each domain are presented in Chang et al. (2016).

To accommodate the different BCDs and implants, small changes were made to the original LiUHead to position the BC transducers and implants for each simulation of a specific BC transducer. The alterations of the FE model and the additions of the BCDs were conducted in the software Hypermesh^©^ (Altair Engineering, Troy, MI, USA). Based on the information for each BCD, the materials of the implanted parts are all modeled as titanium, and the external parts are modeled as plastic. The parameter values for the different structures and parts are shown in Table 1.

**Table 1.** Parameter Values for the Soft Tissue With Different Static Forces and the Bone-Conduction Device Materials and Associated Parameter Values.

Component	Young’s modulus *E* (MPa)	Density ρ (kg/m^3^)	Poisson’s ratio ν	Loss factor η	Element type
Soft tissue	0.7	900	.45	$3\times 10^{-5}\times$	Tetrahedron solid
Soft tissue (3 N)	5	820	.45	$3\times 10^{-4}\times$	Tetrahedron solid
Soft tissue (4 N)	6	815	.45	$3\times 10^{-4}\times$	Tetrahedron solid
Soft tissue (5 N)	7	810	.45	$3\times 10^{-4}\times$	Tetrahedron solid
Soft tissue (6 N)	8	810	.45	$3\times 10^{-4}\times$	Tetrahedron solid
Steal	200,000	7,850	.33	—	Tetrahedron solid
Plastic	32,000	1,190	.35	—	Tetrahedron solid
Titanium	105,000	4,940	.33	—	Tetrahedron solid

*Note.* The loss factor depends on the frequency (*f*) in Hz.

### Simulation Setup

All simulations were computed by the FE solver COMSOL Multiphysics® (COMSOL Inc., Stockholm, Sweden) in the frequency range from 100 to 10k Hz. The frequency resolution was 25 Hz in the range of 100 to 500 Hz, 50 Hz in the range of 500 to 1000 Hz, and 100 Hz in the range of 1 k to 10 k Hz. In the model, three orthogonal directions were defined: *x* direction, which is from the right to the left of the head (medial); *y* direction, which is toward the front of the head (anterior); and *z* direction, which is toward the bottom of the head (inferior; Figure 1).

**Figure 2.**
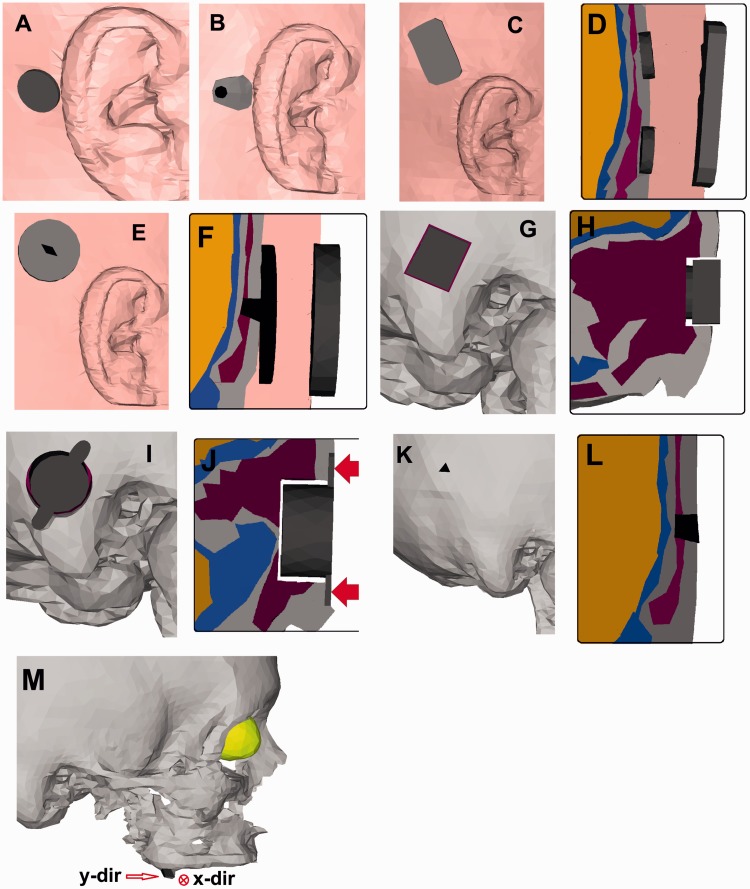
Positions and geometries of the BCDs in the LiUHead used in the simulations. The color scheme is the same as in Figure 1, where pink represents the skin and soft tissue, gray represents cortical bone, purple represents soft bone (diploe), blue represents CSF, and yellow represents the brain tissue. The transducers are illustrated in dark gray and black. (a) Interface of the Radioear B71 on the mastoid skin. (b) The Adhear® interface on the skin behind the pinna. (c) The interface of the Sophono® on the skin behind the ear and (d) a cross section showing the Sophono® interface and implanted magnets. (e) The interface of the Baha® Attract and (f) a cross section showing the Baha® Attract interface and implanted magnet. (g) The BCI transducer placed in the mastoid part of the skull bone and (h) a cross section showing the BCI attaching to the bottom of the hole in the skull bone. (i) The Bonebridge™ transducer with wings attached to the skull bone in the mastoid and (j) a cross section showing the hole in the skull where the Bonebridge™ transducer is positioned, where the two arrows indicate the application of the stimulation force. (l) The stimulation position in the skull bone for the Baha®/Ponto and (l) a cross section showing the implanted screw. (m) The tooth used for stimulation by the SoundBite™ where the two stimulation directions (*x* and *y*) are indicated.

The model is assumed symmetrical at the midline, and all BCDs were located on the right side of the LiUHead (Figure 2). The input to the model was a dynamic force of 1 N applied at the position of the output signal from the BCDs. The BCDs interfaced the LiUHead at the typical position for each device. As a result, the stimulation vectors of the BCDs differed and are noted in Table 2. The cochlear excitation was estimated by the vibration of the cochlear promontories in all three dimensions at both sides. This is not equal to the hearing perception, but the vibration of the cochlear promontory has previously been shown to correlate to the BC sound perception at frequencies between 0.5 and 5 kHz (Eeg-Olofsson et al., 2013). While signal-to-noise considerations limited comparisons of promontory vibration and hearing perception to the 0.5 to 5 kHz frequency range in Eeg-Olofsson et al. (2013), modeling studies of human BC sound perception suggest that the vibration of the bone encapsulating the inner ear dominates the hearing response (Stenfelt, 2015, 2016). Moreover, the cochlear promontory vibrations are used to evaluate all simulated BCDs, and the relative data can be used to compare the efficiency between devices.

**Table 2.** The Stimulation Vectors for Each Bone-Conduction Device.

Position	*x*	*y*	*z*
Radioear B71	.86	.45	−.23
Adhear®	.88	.43	−.22
Sophono®	.98	.22	−.01
Baha® Attract	.95	.29	.05
BCI	.98	.06	−.16
Bonebridge™	.99	−.08	−.15
Baha®/Ponto	.96	.27	.06
SoundBite™ (medial)	.96	.2	−.18
SoundBite™ (frontal)	.22	.93	.28

*Note.*BCI = bone-conduction implant.

On some skin-drive BCDs, the transducer is located outside of the skull and attached to the skin surface by a static force from magnets or a headband. Due to the action of the static force, the soft tissues are deformed leading to an alteration of the material parameter values. To account for this change, the soft tissue material properties between the BCD interface and the skull bone were locally changed. The new values were derived from the data of Cortés (2002) so the mechanical point impedance at the skin surface of the LiUHead equaled the experimental data in the Cortés study for static forces between 3 and 6 N. The soft tissue parameter values as a function of static force are shown in Table 1.

#### Radioear B71

The Radioear B71 (Radioear, USA) is a standard BC transducer for audiometric testing. It is normally positioned on the mastoid skin 20 to 25 mm behind the ear canal opening without touching the pinna. The transducer is held in position by a static force of at least 5.4 N from a headband. Here, the skin parameters for 6 N were used. Only the interface part of the Radioear B71 transducer was included, and it was modeled as a circular plastic plate with 175 mm^2^ surface area and 2 mm thickness (Figure 2(a)), and the stimulation force was equally distributed over the whole area of the plastic plate. It should be noted that the interface size is the same for the newer Radioear B81 transducer, and the analysis conducted here is applicable to that BCD as well.

#### Adhear®

Adhear® (MedEL, Austria) is a new BCD that is attached to the skin with adhesive and does not require a static force. Its position is at the mastoid, similar to the Radioear B71 but closer to the pinna. The interface part is modeled as an equilateral trapezoidal 1-mm thick plastic plate with round edges (Figure 2(b)) where the lengths of the parallel sides are 9 mm and 14 mm, and the midline is 17 mm. The stimulation is applied on a circular area with a diameter of 5 mm at the narrower end of the plate, shown as the dark area in Figure 2(b).

#### Sophono®

The Sophono® (Medtronic, USA) implant system is, at the time of this writing, not commercially available but has a design that is of general interest. This device is modeled with one rectangular plastic plate (35 mm × 20 mm × 4 mm) on the skin and two circular magnets (10 mm diameter and 2.6 mm height) on the skull bone surface interspaced by 10 mm (Figure 2(c) and (d)). The magnets are modeled as titanium for the simulations. The specific position of the center of the implant was reported as approximately 60 mm (O’Niel, Runge, Friedland, & Kerschner, 2014) or 70 mm (Hol et al., 2013) posterior to the external ear canal, at an angle of about 45° posterior and superiorly. In this study, a distance of 60 mm was used, which is close to the Baha® position (see later). In reality, the outer part of the BCD is held in position by the magnetic field resulting in a static force between the BCD and the skull. Here, we model such forces by changing the soft tissue parameters for the volume under the plastic plate (BCD) to values corresponding to a static force of 3 N (Table 1). There is no information on how the vibration unit is connected to the plastic plate in this BCD, and it is modeled by applying the stimulation force as a body load meaning that the entire plastic structure is forced to vibrate as a single unit with the applied stimulation force.

#### Baha® Attract

The Baha® Attract System (Cochlear, Australia; Flynn, 2013) was modeled as a two-part implant where the inner part measures 27 mm in diameter, and a 2.4 mm-thick circular titanium mass attaches to the skull by a centrally located screw measuring 4.3 mm (Figure 2(e) and (f)). For the outer part, a circular plastic plate with a diameter of 29.5 mm and 5.25 mm thickness was used. Like the Sophono®, the Baha® Attract uses a magnetic retention system. Here, it was modeled with a static force of 4 N, and the soft tissue beneath the plastic plate was altered accordingly (Table 1). The input to the implant was on the center of the external plastic plate where the Baha® transducer is normally attached to the Attract system. The attachment area for the Baha® transducer is circular with a diameter of 5 mm and is modeled here as a quadrilateral geometry (dark area in Figure 2(e)) with a similar area (19.7 mm^2^).

#### BC implant

The BC implant (BCI) transducer is surgically placed in the mastoid bone. Here, it is modeled by altering the mastoid geometry by removing skull bone in the model (Figure 2(g) and (h)). The hole made in the model was rectangular with a depth that just fit the transducer, with a space of approximately 1 mm between the bone and transducer on each side. The BCI was modeled as a 14-mm × 12-mm rectangular titanium geometry of 7.4 mm height with a 1 mm thick and 12-mm-diameter cylindrical bottom (Reinfeldt, Håkansson, Taghavi, Fredén Jansson, & Eeg-Olofsson, 2015). Only the circular bottom of the BCI is connected to the skull bone and was here modeled as a rigid coupling, while the space around the implant position was filled with soft tissue. The stimulation force of 1 N was applied to the entire implanted transducer geometry.

#### Bonebridge™

Similar to the BCI, the Bonebridge™ (MedEl, Austria) transducer was also placed inside the mastoid skull bone (Figure 2(i) and (j)). The hole in the mastoid for the Bonebridge™ is circular, and its dimension is 2 mm wider and 1 mm deeper than the implanted transducer. The Bonebridge™ transducer is modeled as a 15.8-mm-diameter titanium cylinder of 8.7 mm height with two lateral rigid wings (Reinfeldt, Håkansson, Taghavi, & Eeg-Olofsson, 2015). There are two holes at the extremity of the wings for screw anchoring of the device into the cortical bone. The Bonebridge™ is attached to the skull bone by the titanium screws (4 mm in diameter) on the two wings of the transducer interspaced by 23.8 mm (Wimmer et al., 2015). The screws are ignored in the current modeling to reduce complexity in the model, and the wings were rigidly attached to the skull bone while the other space surrounding the transducer was filled with soft tissue. The stimulation force was applied to the two wings with 0.5 N on each wing (arrows in Figure 2(j)).

#### Baha®/Ponto

This is the classic position where the BCDs Baha® Connect (Cochlear, Australia) and Ponto (Oticon Medical, Sweden) are positioned with a skin penetrating titanium fixture (Reinfeldt, Håkansson, Taghavi, & Eeg-Olofsson, 2015). In the current modeling, the skin penetrating fixture is ignored, and the stimulation is applied directly to a screw inserted in the skull bone (Figure 2(k) and (l)). The screw is positioned approximately 55 mm behind the ear canal opening in line with the upper part of the pinna and is modeled as 4.3-mm-long steel prism. This setup is the same as position P1 in the study of Chang et al. (2016).

#### SoundBite™

The SoundBite™ hearing system manufactured by Sonitus Medical Inc, US, is no longer commercially available, but applying the BC sound at the teeth is of general interest, and this system is therefore included. The BC transducer of the SoundBite™ system is applied to a molar (Muramatsu, Kurosawa, Oikawa, & Yamasaki, 2013). However, the LiUHead did not include teeth, so a molar in the upper jaw was added to the model (Figure 2(m)). The simulation was applied directly to the added tooth over the tooth surface in two directions: a 14 mm^2^ area in the medial (*x*) direction and a 16 mm^2^ area in the frontal (*y*) direction. The use of two directions is to see whether there are differences in the response depending on the stimulation direction at the tooth.

### Mechanical Point Impedance

One important measure for the different stimulation positions is the mechanical point impedance, *Z*_mech_. This is a measure of the resistance to motion at the stimulation position for the BCD. This is a frequency-dependent complex-valued (magnitude and phase) function and, slightly simplified, on the one hand, a low mechanical point impedance magnitude means a high velocity for a given applied force compared with a high mechanical point impedance magnitude. But, on the other hand, a BCD transducer can most often provide a greater output force when applied to a high mechanical point impedance load than when applied to a low point impedance magnitude load. For optimal performance, the BCD transducer should be designed for its specific application load.

The mechanical point impedance is the quotient between the applied force (*F*) and the resulting velocity (*v*) in the same position:

(1) $$Z_{\text{mech}}=\frac{F}{v}$$

The force (*F*) is integrated over the entire stimulation area, and the response velocity (*v*) is the average velocity of the same area. For most simulations, the force was applied at the interface surface between the LiUHead and the transducer, and *Z*_mech_ is a measure of the skull properties for that specific interface area at that position. However, for two BCDs, Adhear® and Baha® Attract, the force is applied to an adapter on the LiUHead, and *Z*_mech_ includes the load of the adapter as well.

### Stimulation by a BC Transducer

The current evaluation with an equal dynamic force applied to the BCDs’ interfaces indicate the different stimulation positions’ ability to transmit the stimulation force to vibrations at the inner ears that are used as outcome measures. However, a BCD comprises a transducer that converts the electrical signal (supplied mostly by a battery) to an output force. In most BCDs, this voltage to the transducer limits the amount of excitation possible, the maximum power output. Therefore, a model of a BC transducer is included here to evaluate the different BCDs when a voltage of 1 V is applied to a BC transducer attached to the stimulation position of the BCDs in Figure 2.

**Figure 3.**
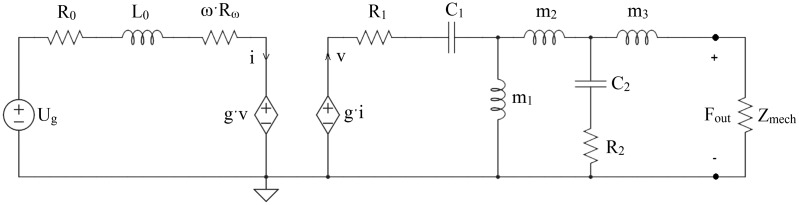
A lumped-element model of the Radioear B71 transducer and the Baha®/Ponto transducer. A detailed description of the included elements can be found in the text and the values of the elements are given in Table 3.

For most BCDs, the specific characteristics of the BC transducers are not provided by the manufacturers. In this study, two BC transducers will be used: one for the Radioear B71 and the other is a typical Baha®/Ponto BC transducer. Both BC transducer models have the same topology, but the Radioear B71 model includes the house resonances in the casing while the Baha®/Ponto model assumes that the transducer output is coupled rigidly to the place of stimulation. The model for both transducers is shown in Figure 3 as a lumped-element electromechanical system. The parameters of the Radioear B71 are from Lundgren (2011), and the parameters of the Baha®/Ponto are calculated from experimental data on input–output characteristics of a Baha® transducer measured in our laboratory. In this model, *ω* is the angular frequency, and the other parameter values are shown in Table 3. The parameter *F*_out_ is the force applied to the models of the BCDs in Figure 2.

**Table 3.** The Parameter Values for the Transducer Model of the Radioear B71 and the Baha®/Ponto Shown in Figure 3.

	Radioear B71	Baha/Ponto
*U_g_* (V)	1	1
*R*_0_ (Ω)	3.4	8
*L*_0_ (mH)	0.86	0.86
$R_{\omega }$ (Ω)	0.0004	0.0004
*g*	3.3	4
*m*_1_ (kg)	0.01633	0.015
*C*_1_ (µm/N)	4.055	3
*R*_1_ (Ns/m)	1	40
*m*_2_ (kg)	0.00256	0.0015
*C*_2_ (µm/N)	1.3	0.1
*R*_2_ (Ns/m)	2	20
*m*_3_ (kg)	0.0035	0

The model converts an electrical input to mechanical force output. The input voltage is provided by *U_g_* in the model, while *R*_0_ and *L*_0_ models the resistance and inertance of the transducer coil, and *R_ω_* models the magnetic losses in the transducer. The conversion from the electrical current *i* to the mechanical velocity *v* is accomplished by the gyrator *g*. The resistance *R*_1_ and the compliance *C*_1_ are the damping and compliance of the transducer suspension, while the mass *m*_1_ represents the mass of the transducer. The T-branch including *m*_2_, *m*_3_, *R*_2_, and *C*_2_ is the model of the housing for the Radioear B71 transducer and the connection unit for the Baha®/Ponto transducer. For the Radioear B71 transducer, the mass of the casing is divided between the mass that moves with the load (*m*_3_) and the rest of the mass (*m*_2_), and the compliance *C*_2_ forms the house resonances with the *m*_2_ and *m*_3_ masses. For the Baha®/Ponto transducer, *m*_2_ represents the mass of the connection part of the transducer, and *C*_2_ and *R*_2_ are the compliance and damping at the interface of the transducer attachment. The latter forms a high-frequency resonance that is at a frequency above the frequency range investigated here. The mass *m*_3_ is not included in the Baha®/Ponto transducer.

## Results

In this study, the stimulation positions were in accordance with the excitation methods of each BCD. The direct-drive BCDs, including the oral device, were rigidly connected to the skull bone, while the skin-drive BCDs were rigidly coupled to the plastic part on the skin. For each BCD, the acceleration responses were obtained at both the ipsilateral and contralateral cochlear promontories in three perpendicular directions.

### Mechanical Point Impedance

**Figure 4.**
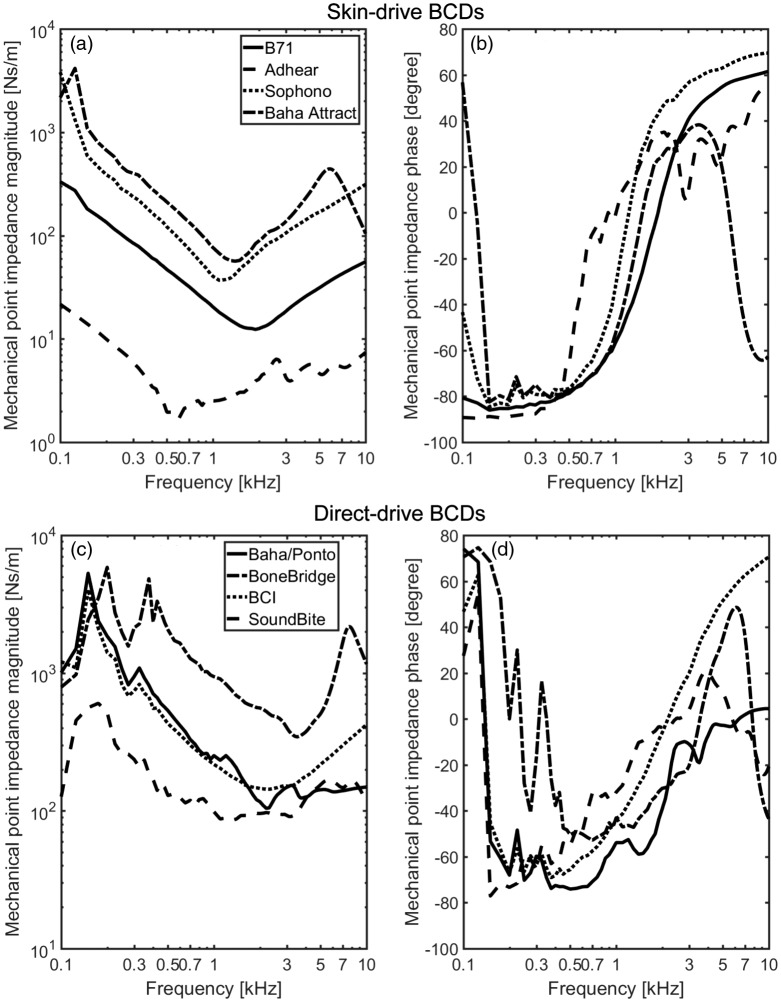
The mechanical point impedance for the BCDs, shown as magnitude (left panels) and phase (right panels). (a) and (b) skin-drive BCDs and (c) and (d) direct-drive BCDs. BCI = bone-conduction implant.

The magnitudes and phases of the mechanical point impedance results are displayed in Figure 4. The frequency axis is logarithmic, and the results cover the frequency range of 0.1 to 10 kHz. The results are grouped according to the stimulation method of the BCDs.

#### Skin-drive BCDs

The mechanical point impedances of the Radioear B71, Sophono®, Adhear®, and Baha® Attract, as magnitude and phase, are shown in Figure 4(a) and (b). All mechanical point impedance curves show the same tendencies. At low enough frequencies, the mechanical point impedance is determined by the entire mass of the head. The LiUHead has a total mass of 4.96 kg which would cause an impedance magnitude of approximately 3.1 × 10^3^ Ns/m at 0.1 kHz. The influence from the head mass on the mechanical point impedance is visible for the Sophono® and Baha® Attract at the lowest frequencies in Figure 4(a) and (b). For the Radioear B71 and Adhear®, the attachment stiffness is too low for the impedance of the head mass to be visible for those two at 0.1 kHz.

At frequencies above 0.1 kHz for the Radioear B71 and Adhear® and above 0.15 kHz for the Sophono® and Baha® Attract, and up to the resonance frequency formed by the mass and stiffness in the soft tissue, the magnitudes fall with frequency indicating a stiffness-controlled system. Above this resonance frequency that appears between 0.6 and 2.0 kHz for the different BCDs, the magnitudes of the mechanical point impedance increase with frequency, suggesting a mass-controlled system. The phases also increase with frequency above 0.15 kHz from negative values close to −90° to positive values approaching 60°. The exception is the results of the Baha® Attract that have a second resonance at around 6 kHz, above which the magnitude falls with frequency and the phase drops to −60°. This second resonance is caused by a resonant mode in the circular plate of the Baha® Attract.

The resonance frequencies and the magnitudes of the mechanical point impedance differ between the BCDs. The Adhear® had the lowest resonance frequency at approximately 600 Hz, and the magnitude is the lowest of all skin-drive BCDs with around one order of magnitude lower than the impedance magnitude of the Radioear B71. The resonance frequency of the Sophono® is around 1.1 kHz, the Baha® Attract around 1.4 kHz, and the Radioear B71 about 2 kHz. This resonance is a series resonance of the soft tissue mass that moves with the BCD and the compliance of the soft tissue seen from the BCD. Both of these depend on the interface area; a greater area results in a larger mass and stiffer connection compared with a smaller area.

The effect of interface area between BCD and skin is also visible in the impedance magnitudes. The Baha® Attract and Sophono® had the highest impedance magnitudes, around 0.5 to 1 order of magnitudes greater than the Radioear B71. As stated earlier, a greater area means a higher stiffness and a larger mass to move, resulting in a greater impedance magnitude.

#### Direct-drive BCDs

The mechanical point impedances of the four direct-drive BCDs, Baha®/Ponto, BCI, Bonebridge™, and the SoundBite™, are shown in Figure 4(c) and (d). Here, the results from the SoundBite™ are only shown with the excitation direction similar to the three other direct-drive BCDs (*x*-direction), as the result obtained in the perpendicular direction was found similar.

As explained earlier, the behavior at the lowest frequencies is caused by the mass of the head. It manifests itself as a frequency-dependent magnitude increase accompanied by a positive phase. The low-frequency resonance is a parallel resonance caused by the entire mass of the head and the stiffness of the skull bone at the stimulation position. This first resonance frequency of the direct-drive BCDs lay between 0.125 and 0.2 kHz, where the resonance frequency appears at 0.15 kHz for the Baha®/Ponto and the BCI, 0.2 kHz for the Bonebridge™, and 0.125 kHz when the SoundBite™ was stimulated in the medial (*x*) direction. There is a smaller second resonance at 0.325 kHz for the Baha®/Ponto and BCI, but for the Bonebridge™, the second resonance at 0.375 kHz is similar in magnitude to the first. The mechanical point impedance magnitude of the Bonebridge™ was approximately 0.5 order of magnitude greater than the other two direct-drive BCDs, while the impedance magnitude of the SoundBite™ was around 0.5 order of magnitude lower.

Above the low-frequency resonances, the magnitudes of the mechanical point impedances decrease with frequencies until approximately 2 to 3.5 kHz, indicating a stiffness-dominated system. The corresponding phases show negative values also indicating stiffness dominance. However, at frequencies above 2 to 3.5 kHz, the trends of the impedances become different where the magnitudes of the Baha®/Ponto and SoundBite™ start to flatten out and the phases approaches zero. But for the BCI and the Bonebridge™, the magnitudes of the impedances increase with frequency and the phases increase to 50° to 70°. At the highest frequencies, above 7.5 kHz, the impedance magnitude of the Bonebridge™ declines with frequency, and the phase drops to about −50°. This behavior seems similar to the high-frequency impedance of the Baha® Attract in Figure 4(a) and (b). However, the mechanisms for the high-frequency resonance differ where the plate mode is the origin for the Baha® Attract resonance while the Bonebridge™ high-frequency resonance is likely due to the attachment by two spatially separated screws.

The higher point impedance magnitudes of the Bonebridge™ compared with the other BCDs are most probably also caused by the differences in attachment. The Bonebridge™ is anchored in the bone at two spatially different positions resulting in a stiffer loading and also more mass that vibrate with the excitation at higher frequencies compared with a single position attachment.

### Accelerance Responses

**Figure 5.**
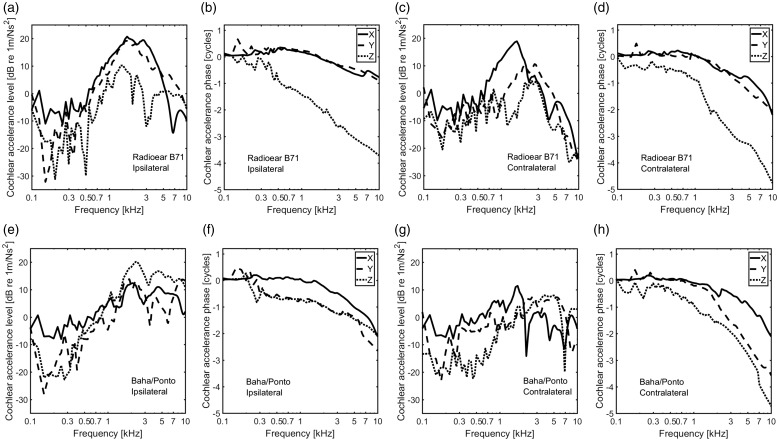
The accelerance (acceleration/force) at the cochlear promontories from the simulations of two typical BCDs, the Radioear B71 (a to d) and the Baha®/Ponto (e to h). The top row presents the results with the Radioear B71, and the bottom row presents the results with the Baha®/Ponto. Solid line: *x* direction, dashed line: *y* direction, dotted line: *z* direction.

The response accelerations were obtained at the cochlear promontory on each side of the head, in three perpendicular directions (*x*, *y*, and *z* direction as shown in Figure 1) for all BCDs. Although different BCDs show different cochlear promontory responses, the same type of BCDs displayed similar overall tendencies. Therefore, only the accelerances of the Radioear B71 and the Baha®/Ponto, considered typical BCDs of the skin-drive and the direct-drive BCDs, are presented in Figure 5 as the level and phase for all three vibration directions. The accelerance is defined as the response acceleration divided by the input force. Solid, dashed, and dotted lines display the responses in the *x*, *y*, and *z* directions, respectively. The results from the Radioear B71 are presented in Figure 5(a) to (d) (top row), and the results from the Baha®/Ponto are presented in Figure 5(e) to (h) (bottom row).

The level responses shown in dB re 1 m/Ns^2^ should be interpreted as the acceleration level in m/s^2^ in the three directions, when the excitation force is 1 N at the stimulation position with a direction according to Table 2. The accumulation of phase indicates the time delay, which is due to the vibratory wave transmission in the head between the attachment positions of the BCDs and the cochlear promontories. For both BCDs, at low frequencies, approximately below 0.5 to 0.6 kHz, the accelerance levels are between −10 and 0 dB re 1 m/Ns^2^ in the main direction of the stimulation and somewhat lower in the other directions. The related phases also show a flat tendency close to either 0 or ±0.5 cycles. At those low frequencies, the head approximates rigid body motion and moves as a whole and the translational and rotational inertia determine the responses.

Above the frequencies of rigid body motion, the accelerance levels in all directions tend to increase with frequency, and all the phases decrease with frequency. Almost all magnitudes of the BCDs have a maximum value at a frequency between 1 kHz to 4 kHz. For the Radioear B71, the maximum level is around 20 dB re 1 m/Ns^2^ on the ipsilateral side and 15 to 20 dB re 1 m/Ns^2^ on the contralateral side. For the Baha®/Ponto, the maximum level is 20 dB re 1 m/Ns^2^ on the ipsilateral side and 10 dB re 1 m/Ns^2^ on the contralateral side. At frequencies above the frequency of the level maximum, the levels decrease with frequency. Here, the levels of the skin-drive BCD (Radioear B71) drop more and more rapidly than the direct-drive BCD (Baha®/Ponto). Moreover, the levels and phases of the cochlear promontory vibrations at the contralateral side decrease more than at the ipsilateral side.

To facilitate easier comparison between the results with stimulation at the different positions, the accelerance magnitudes in all three dimensions are computed as a composite level, here termed the total accelerance (*A*_TOT_). The total accelerance is computed as the square root of the sum of the components multiplied with their complex conjugates, see Equation (2).

(2) $$A_{\text{TOT}}=\sqrt{A_{x}\times A_{x}^{*}+A_{y}\times A_{y}^{*}+A_{z}\times A_{z}^{*}}$$

**Figure 6.**
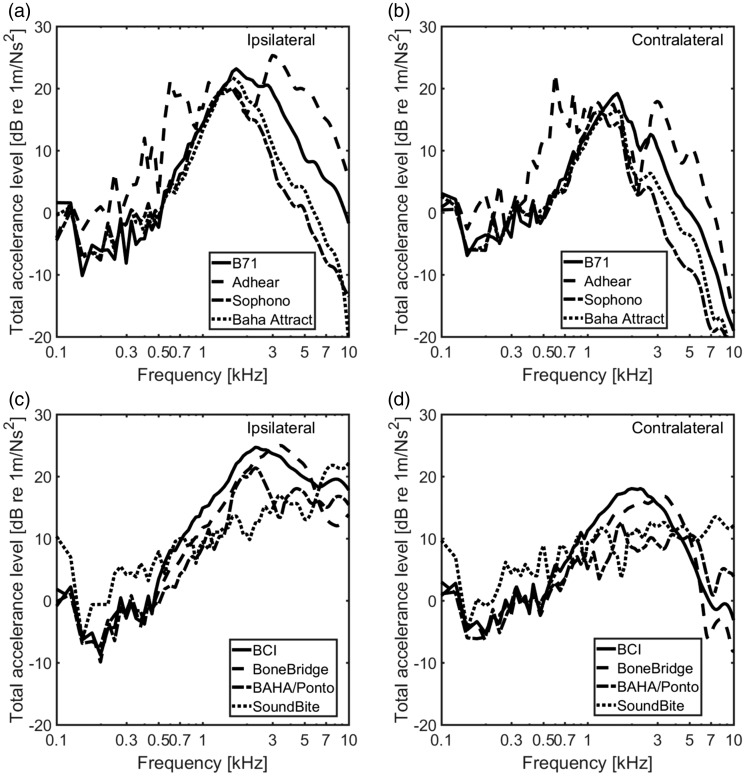
The level of the total accelerance, computed as the square root of the sum of the squared accelerances in the three orthogonal directions at the cochlear promontories. The results from the skin-drive BCDs are presented in the top panels with ipsilateral accelerance levels (a) and contralateral accelerance levels (b) while the direct-drive BCDs are presented in the bottom panels with ipsilateral accelerance levels (c) and contralateral accelerance levels (d). BCI = bone-conduction implant.

Here, *A_x_*, *A_y_*, and *A_z_* are the accelerances in the *x*, *y*, and *z* directions, respectively, and * indicates the complex conjugate. This measure contains only the magnitude information ignoring the phase data. The total accelerances of the BCDs are displayed in Figure 6. The results are grouped as the skin-drive BCDs (Figure 6(a) and (b)) and the direct-drive BCDs (Figure 6(c) and (d)). The maximum levels at the cochlear promontory with a 1 N dynamic force applied at the stimulation positions of the BCDs appear at frequencies between 1 and 4 kHz. The total accelerance at the ipsilateral side shows a slightly higher level than at the contralateral side. At frequencies above 3 kHz, the total accelerance levels of the cochlear promontory from the skin-drive BCDs decrease faster than those from the direct-drive BCDs, and the total accelerance levels at the ipsilateral side show less decrease than those from contralateral side.

At frequencies below 2 kHz, the cochlear promontory vibration levels from the skin drive BCDs are similar except for the Adhear® system that shows 5 to 10 dB greater values in an irregular fashion. This result can be attributed to the low mechanical point impedance at the skin surface for that system (Figure 4(a)) resulting in greater excitation velocities compared with the other BCDs when the same stimulation force is applied. At higher frequencies, the Baha® Attract and Sophono® show results that are approximately 10 dB worse compared with the Radioear B71, while the results for the Adhear® are 5 to 10 dB greater than the Radioear B71 results.

The total accelerance levels from the direct-drive systems are more similar than those from the skin-drive BCDs and are generally within 5 to 10 dB of each other (Figure 6(c) and (d)). At the ipsilateral side, the total accelerance level with the SoundBite™ seems to be slightly worse than the total accelerance levels from the other BCDs, while it shows 10 to 20 dB greater response level at the contralateral side for the highest frequencies investigated.

### Stimulation by a BC Transducer

All the earlier results were the cochlear promontory acceleration responses when the stimulation was a dynamic force of 1 N. However, in reality, stimulation is the vibration output from a transducer. For an equal input voltage, the output from the transducers of the BCDs differs due to the different mechanical point impedances loading each BCD. To study the cochlear promontory vibration responses with the same electrical stimulation level of the BCDs, the transducer model shown in Figure 3 was used. In this study, the Radioear B71 used the transducer model with the parameters of the Radioear B71 and the other BCDs all used the parameters for the Baha®/Ponto transducer (the parameter values are given in Table 3).

**Figure 7.**
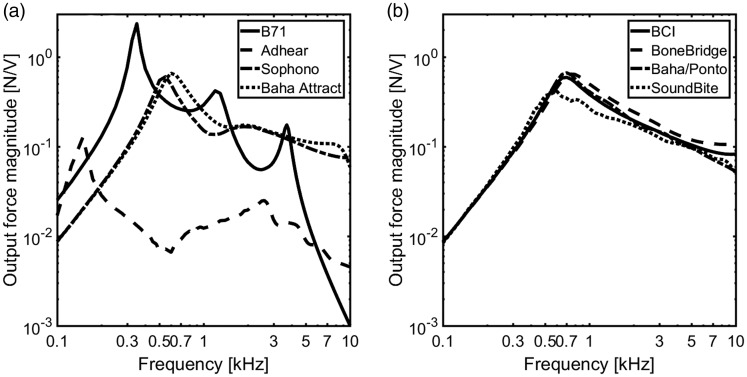
The output force magnitudes of the transducer models in Figure 3 excited by 1 V and loaded according to the impedances for the specific BCD in Figure 4. BCI = bone-conduction implant.

The output force levels of the transducers loaded with the mechanical impedances in Figure 4 and with an electric input of 1 V are displayed in Figure 7, grouped as skin-drive BCDs (Figure 7(a)) and direct-drive BCDs (Figure 7(b)). The output forces of the Sophono®, Baha® Attract, and all the direct-drive BCDs are similar, within 6 dB. The forces of those BCDs increase with frequency with a maximum between 550 and 750 Hz and then decrease about half an order of magnitude up to 10 kHz. However, the output forces of the Radioear B71 and Adhear® show different results. The output force obtained from the Radioear B71 has three peaks, and the maximum level is found at 350 Hz, which is the highest output level of all the BCDs. The other two peaks are at 1.2 kHz and 3.6 kHz, and noticeable is the great loss of output force above the third resonance frequency with an approximately slope of −40 dB/octave. The Adhear® has the lowest level of the output forces overall, around one order of magnitude less than the others.

**Figure 8.**
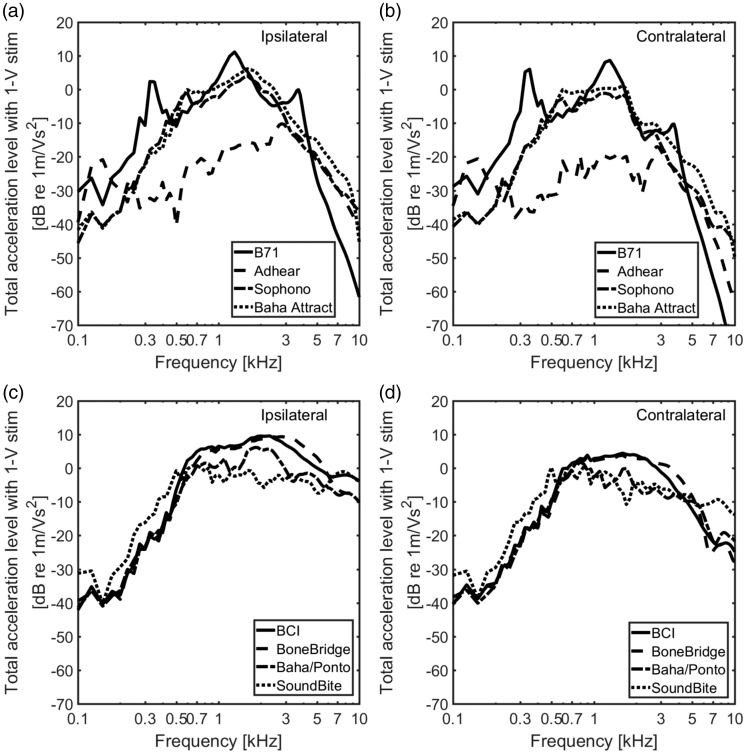
The total acceleration levels (square root of the sum of the squared cochlear promontory orthogonal components) of the BCDs when the stimulation is 1 V to the transducer model in Figure 3. The skin-drive BCDs’ ipsilateral total acceleration levels (a) and the contralateral total acceleration levels (b) are presented, while the direct-drive BCDs’ ipsilateral total acceleration levels (c) and the contralateral total acceleration levels (d) are presented. BCI = bone-conduction implant.

When the output forces of the BCDs computed earlier were applied to the model for each BCD (combining Figures 6 and 7), the levels of the total acceleration levels with 1 -V stimulation to each BCD are presented in Figure 8 in a way similar to Figure 6. Overall, the voltage-driven total accelerations of the direct-drive BCDs are smoother than the skin-drive BCDs, and the levels on the contralateral side are lower than on the ipsilateral side. Compared with the total accelerance levels displayed in Figure 6, the differences of the total acceleration levels between the direct-drive BCDs and the skin-drive BCDs became less from 500 to 3 kHz when the transducers were incorporated, except for the Adhear®. The skin-drive BCDs all show worse high-frequency results than the direct-drive BCDs, and the two BCDs that differ most from the others are the Radioear B71 and the Adhear® systems. The Radioear B71 has a different transducer model which incorporates a lower first resonance and resonances in the housing thereby giving better low-frequency results and worse high-frequency results. The Adhear® BCD interfaces a low impedance resulting in a low stimulation force out of the transducer, and it is only at frequencies above 3 kHz that this system shows comparable results to the other skin-drive devices.

## Discussion

In this study, according to the actual methods of positioning or implantation, the BC-related parts from eight different BCDs were modeled and added to the LiUHead. The FE method has its own limitation: The size of the mesh as well as the included domains and parameters could affect the accuracy of the simulation results. But with consideration to the hardware requirement and the simulation time, the LiUHead presents results with reasonable levels of accuracy (Chang et al., 2016). For the modeling of the BCDs, only the external structure of the BC-related part was added to the LiUHead. The material parameters and the details of the structure might also affect the accuracy. However, this study facilitates comparison of the responses from different BCDs in the same head, which minimize individual differences. It should be noted that the mechanical response of the LiUHead is fitted to average data from multiple studies on BC sound transfer functions in the human head, and the response in a single head can differ from the average responses that are presented in this study.

The vibration responses from each BCD were obtained as the acceleration on both sides of the cochlear promontories in three perpendicular directions in the frequency range from 0.1 to 10 kHz. The results displayed the cochlear promontory vibration responses for different BCDs with the same output force from the transducer (here chosen to be 1 N). Moreover, with the mechanical point impedances and the lumped-element model of the transducers, the results with the same electric input to the transducer were calculated, which indicate the responses for the same sound pressure level at the microphone of the BCDs when the gain of the BCDs are the same. Although the vibration responses of the cochlear promontory are not equal to the sensation level of hearing, the results could indicate the cochlear stimulation from the different BCDs, and the inter-BCD levels can be interpreted as the differences in ability to provide a sensational level for the BC sound.

With all these limitations, the differences reported between the BCDs should be interpreted as general and a different result can be obtained in a specific person. Small, frequency limited differences that are reported in this study should therefore not be interpreted as significant. However, the general trends from the simulations can be translated to clinically significant results, such as overall lower cochlear vibrations or a high- or low-frequency dependent increase or decrease of the cochlear vibration.

### Mechanical Point Impedance

Although several clinical and experimental studies of the skin-drive BCDs have been presented (Flynn, 2013; Mulla, Agada, & Reilly, 2012), the mechanical point impedance measured in living humans still requires several more reports. The exception is the mechanical point impedance at the mastoid for the Radioear B71 interface that has been thoroughly investigated. One of the most thorough studies on this impedance is the report by Flottorp and Solberg (1976). This impedance has also been mimicked, without perfect match, in the artificial mastoids used for audiometric calibrations.

Cortés (2002) measured the mechanical point impedance for the Radioear B71 interface at the mastoid in 30 participants with a static force of about 5.9 N. As the LiUHead skin impedance is independent of the static force, the soft tissue parameters were refitted to correspond to those results. For most of the skin-drive BCDs, the BCDs are attached to the surface of the skin using static force. Khanna, Tonndorf, and Queller (1976) reported that an increase in the static force between the transducer and the skin-covered skull improved the BC thresholds, which indicated that the static force influences the BC transmission. Cortés (2002) reported the mechanical point impedance of the skin in one subject with different static forces showing that the static force influenced the mechanical point impedance. According to results in Cortés, the material parameters of the soft tissue were changed with the different static forces (Table 1). With increasing static force, the mechanical point impedance stiffness increased, and the resonance frequency of the mechanical point impedance became higher.

The skin-drive BCD Sophono® was simulated with a static force of 3 N, and the parameter values of the soft tissue for the Sophono® simulation were those given in Table 1 for 3 N. The area of the Sophono® is about 4 times larger than the interface area of the Radioear B71, and the impedance magnitude of the Sophono® was also around 4 times higher than that of the Radioear B71. The Baha® Attract was applied with a static force of 4 N, and the size of the Baha® Attract interface is also about 4 times larger than that of the Radioear B71. But the major difference between the Baha® Attract and the Radioear B71 or the Sophono® was that the stimulation position was on a small area on the surface of the Baha® Attract plastic plate. This means that the mass and the vibration pattern of this plate is included in the impedance computations. For example, the decline of the mechanical impedance magnitude at frequencies above 6 kHz was caused by a bending motion of the plate reducing its effective stimulation area at the high frequencies.

The mechanical impedance of the Adhear®, which is the only skin-drive BCD attached without a static force, shows a significant lower magnitude and resonance frequency compared with the other skin-drive BCDs. The stimulation position of the Adhear® was similar to the Baha® Attract, on a small surface of the plastic part interfacing the BCD and the skin. The fluctuation in both the magnitude and phase of the mechanical impedance above 2 kHz indicated a complex vibratory motion of this thin plastic part. The thickness of the plastic interface for the Adhear® (1 mm) was less than for the Baha® Attract (5.25 mm), which resulted in more and complex vibratory modes at the high frequencies for the Adhear® interface.

The direct-drive BCDs were implanted into the skull or fixed to the bone by screws and were simulated with the stimulation applied at the skull bone. Previous studies with similar measurements in cadaver heads (Eeg-Olofsson, Stenfelt, & Granström, 2011; Eeg-Olofsson, Stenfelt, Tjellström, & Granström, 2008; Stenfelt & Goode, 2005b) or living subjects (Håkansson, Carlsson, & Tjellström, 1986) show the mechanical impedance of the Baha®/Ponto to be similar with the current data. The main difference between the mechanical impedances of the Baha®/Ponto and BCI above 3 kHz is caused by the position, size, and implanted methods. The mechanical impedance of the Bonebridge™, however, displayed two low-frequency resonant peaks and a resonance at 7 kHz. This behavior is attributed to the two stimulation positions at both wings of the implanted transducer (arrows in Figure 2(j)).

Stenfelt and Håkansson (1999) reported the mechanical point impedance of the teeth obtained from three living participants. However, the mechanical impedance was measured when the participants used their upper and lower incisors to bite on a plastic adaptor coupled to an impedance head, and the results were more similar with the mechanical point impedance of the skin-drive BCDs. The mechanical point impedance for the SoundBite™ was obtained from a molar with opened jaw. Therefore, the simulation results showed disagreement with the experimental data of Stenfelt and Håkansson (1999) and more similarity to the mechanical point impedance of the direct-drive BCDs.

The mechanical point impedances presented here are technical in their nature. However, the mechanical point impedances provide valuable information for the different BCDs. For example, the very low mechanical point impedance magnitude for the Adhear® device shows that although it had good accelerance transmission as indicated in Figure 6(a), the low impedance led to low output from the transducer model in Figure 7(a) resulting in an overall lower performance than other BCDs as indicated in the vibratory results presented in Figure 8(a). The mechanical point impedances obtained from the LiUHead are novel for some of the BCDs.

### Vibration of the Cochlea

There have been several reports of different BCDs in clinical and experiment settings during the last two decades. However, most investigations presented the hearing thresholds or sensitivity as the outcome measure and not the vibration response from the cochlear promontory as in this study. The Baha®/Ponto, as a percutaneous Baha®, was the first available direct-drive BCD and probably the most powerful BCD device today (Reinfeldt, Håkansson, Taghavi, & Eeg-Olofsson, 2015). There are several experimental data sets of the Baha®/Ponto as the vibration response of the cochlear promontory obtained from humans, cadaver, or living (Eeg-Olofsson et al., 2008, 2011, 2013; Sim et al., 2016; Stenfelt & Goode, 2005b). Therefore, the Baha®/Ponto has often been used for comparison with other BCDs. Moreover, the LiUHead was validated with experimental data measured in cadavers and living humans with the stimulation at the Baha®/Ponto position (Chang et al., 2016). In this study, the results of the Baha®/Ponto are used to compare the results from the other BCDs, discussed later. Moreover, as the audiometric BCD, the Radioear B71 with a headband or softband is also used as the gold standard when assessing other BCDs.

Figure 5 only shows the results from the Radioear B71 and the Baha®/Ponto. Those two typical BCDs could represent the characteristics of both skin-drive and direct-drive BCDs. At most frequencies, the results in Figure 5 show the highest level of the accelerance in the direction coinciding with the stimulation direction. This direction also showed the least phase accumulation, which indicated the least time delay. However, sensitivity of BC perception based on the direction of the vibration at the human cochlea is currently unknown, and an approximation of the sound perception is based on the vibrations in all directions, here computed as the total accelerance (Equation (2), Figure 6). The total accelerances of the direct-drive BCDs show similar results with the skin-drive BCDs at frequencies from 0.5 to 3 kHz, but at higher frequencies, the response magnitudes were greater with the direct-drive BCDs compared with skin-drive BCDs.

**Figure 9.**
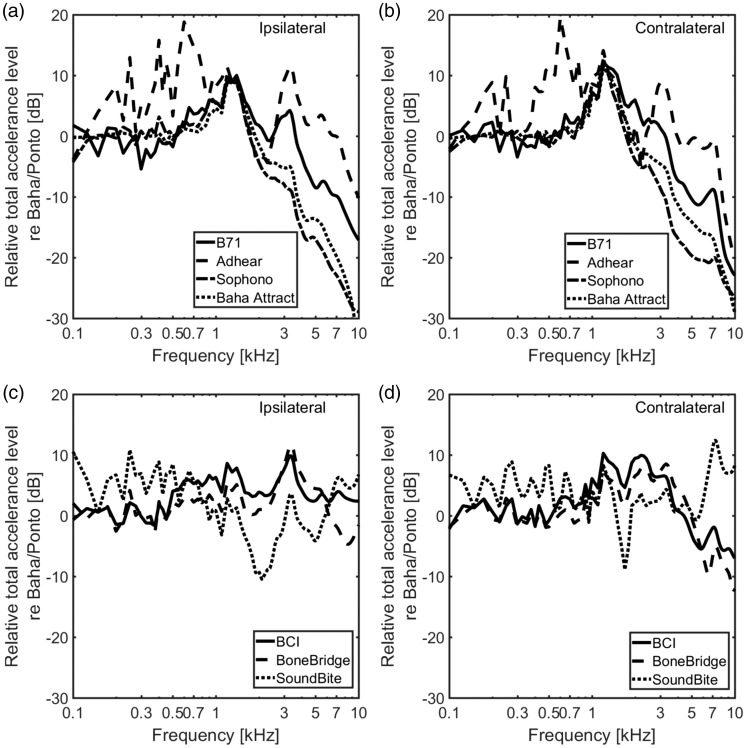
The level of the relative total accelerance computed as the ratio of the accelerance between the BCDs and the BAHA/Ponto from Figure 6. The skin-drive BCD results at the ipsilateral cochlear promontory (a) and contralateral cochlear promontory are shown (b) while the direct-drive BCD results at the ipsilateral cochlear promontory (c) and contralateral cochlear promontory (d) are shown. BCI = bone-conduction implant.

To facilitate comparison between the BCDs, the total accelerances for the BCDs were related to the total accelerance of the Baha®/Ponto, displayed in Figure 9. The zero level indicates a result equal to the Baha®/Ponto when the stimulation is a force of 1 N. According to the comparisons in Figure 9(c) and (d), all the direct-drive BCDs are similar to the Baha®/Ponto, the results are primarily within 5 dB except at a few frequencies. The skin-drive BCDs display similar results to the Baha®/Ponto at low frequencies, and up to 10 dB better results around 1 kHz (Figure 9(a) and (b)). However, above 1.5 kHz, the total accelerances obtained from the skin-drive BCDs at the ipsilateral side are between 10 and 36 dB worse than for the Baha®/Ponto. This indicates ineffective BC transmission compared with the Baha®/Ponto at high frequencies.

### Effect of the Transducer

A more valid comparison between the BCDs is the ability to provide a hearing sensation from an equal electric stimulation level. Therefore, the transducer models for the Radioear B71 and BAHA/Ponto were used. The output forces from the BCDs using the transducer models with 1 -V input are shown in Figure 7. One interesting finding in Figure 7 is the small differences in output force level among the BCDs, the direct-drive BCDs are within 5 dB, and the Baha® Attract and Sophono® devices are within 6 dB of the Baha®/Ponto output. This can be explained by the magnitude of the output impedance of the Baha®/Ponto transducers: As long as this magnitude is significantly lower than the load impedance, most of the generated force is delivered to the load (head). It is only when the load impedance becomes very low, as in the case of the Adhear® device, that the force delivered to the load becomes significantly lower. For the Radioear B71, the explanation is that the house resonances at higher frequencies affect the output force that is different from the Baha®/Ponto transducer.

Based on the output forces in Figure 7, the total accelerances with 1 V to the transducer were computed and presented in Figure 8. It should be noted that the models are linear, and the input voltage can be chosen arbitrarily. In reality, the transducers produce nonlinear distortions that can affect the output, especially at higher stimulation voltages. Compared with the results shown in Figure 6, most accelerances in Figure 8 have similar levels between 0.5 and 3 kHz, which indicated that the different point impedances leveled out the BC stimulation between the different BCDs.

It should be noted that the model in Figure 3 only represents the output transducer of a BCD, while the microphone, amplifier, and other electronics are ignored. This means that the sensitivity of the microphone, the gain of the amplifier, and settings of the filters that also influences the output of the BCD are not included in the current simulations. However, the maximum output of a BCD, the maximum power output in hearing level, is determined by the voltage to the transducer, the transducer itself, and the BC transmission from the attachment position to the inner ear (van Barneveld, Kok, Noten, Bosman, & Snik, 2018). Consequently, the estimations of the outputs in Figure 8 are the maximum output of a BCD with a 1 -V battery. Therefore, if a higher gain is applied to a BCD to reach the hearing threshold, the maximum power output is reached at a lower hearing level than if a lower gain is applied (van Barneveld et al., 2018). Hence, the differences in Figure 8 are estimations of the differences in dynamic ranges between the BCDs.

**Figure 10.**
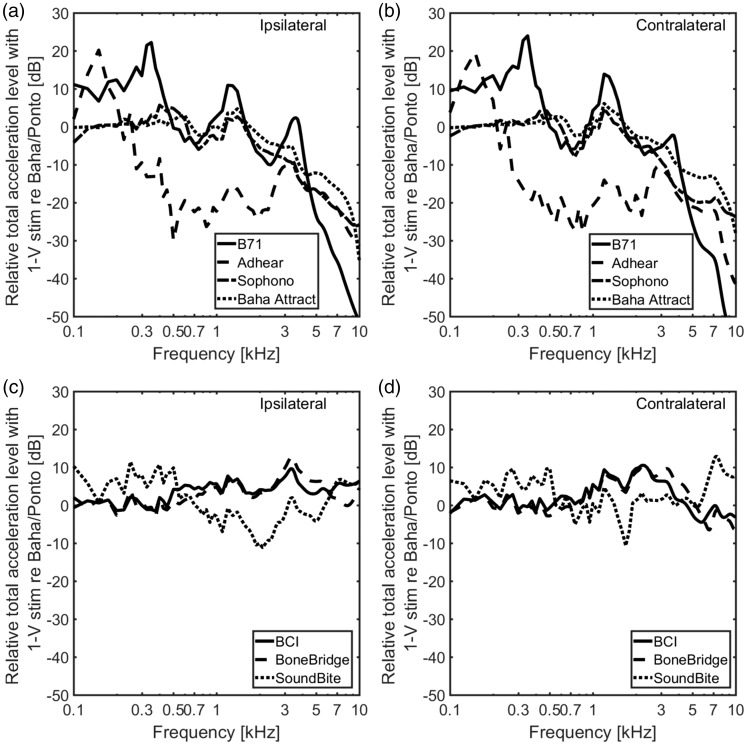
The level of the relative total acceleration with transducer stimulation computed as the ratio of the acceleration with 1 V stimulation between the BCDs and the BAHA/Ponto from Figure 8. The skin-drive BCD results at the ipsilateral cochlear promontory (a) and contralateral cochlear promontory (b) are shown, while the direct-drive BCD results at the ipsilateral cochlear promontory (c) and contralateral cochlear promontory (d) are shown. BCI = bone-conduction implant.

To facilitate comparison between the BCDs with electric stimulation as input, Figure 10 shows the results of the BCDs compared with the Baha®/Ponto. In Figure 10, the relative total accelerance levels with 1 -V stimulation show that, except for the Radioear B71 and the Adhear®, all BCDs have similar performance at frequencies up to 2 kHz, and the direct-drive BCDs show results within 10 dB for the entire frequency range. Previously, the Baha®/Ponto has been reported as superior to skin-drive BCDs, such as older devices similar to the Sophono®, but with other dimensions and attached with a headband, and the Radioear B71 or Baha® Attract (Håkansson, Tjellström, & Rosenhall, 1984; Heywood, Patel, & Jonathan, 2011; Stenfelt & Håkansson, 1999; Verstraeten, Zarowski, Somers, Riff, & Offeciers, 2009; Zarowski, Verstraeten, Somers, Riff, & Offeciers, 2011). The simulations also present results corroborating the conclusions of those studies. Figure 10(a) and (b) reveals that the Radioear B71 gives better cochlear promontory vibration levels than the Baha®/Ponto at the lowest frequencies but is, except for a couple of frequency ranges, less efficient at the middle and high frequencies.

Hol et al. (2013) reported similarities between the Sophono® and the percutaneous Baha®/Ponto and concluded that the Baha®-based outcome was slightly better, especially in the high frequencies. The Sophono® has also been compared with Baha® on a headband, which is similar to the Baha® Attract system. Such studies have indicated similar performance between the Sophono® system and the Baha® on a headband (Denoyelle et al.*,* 2015; O’Niel et al.*,* 2014). Powell, Rolfe, and Birman (2015) compared the performance in six subjects fitted with the Sophono® system with six other subjects fitted with the Baha® Attract system and found them comparable. This was also found in this study; the Baha® Attract and the Sophono® systems result in comparable vibration levels of the cochlear promontories (Figures 6(a) and 8(a)). When the maximum power output was estimated for the Sophono®, Baha®, and Baha® Attract, the Sophono® was 18 to 30 dB below the Baha® system at frequencies between 0.5 and 2 kHz except at 1.5 kHz where they were similar (van Barneveld et al., 2018). That is significantly different from the above-mentioned comparisons that were based on hearing thresholds and also different from the estimations in Figure 10. One reason for this difference is that different output transducers are used in the BCDs indicating that a transducer with a resonance frequency around 1.5 kHz is used in the Sophono® system, while a transducer with a resonance closer to 1 kHz is used in the Baha® system. This study shows that using the Sophono® method of BC stimulation, similar cochlear vibrations to the Baha®/Ponto can be achieved up to 2 kHz, but worse results are expected at higher frequencies (Figure 10(a) and (b)).

Kurz, Flynn, Caversaccio, and Kompis (2014) measured hearing thresholds with the Baha® Attract by attaching the Attract system to the Baha® implant and adding artificial skin between the inner magnet and the outer plastic part of the Attract system. When the results were compared with attaching a Baha® directly to the implant, the Attract system was found to attenuate the vibrations at the higher frequencies. This is similar to this study where the Baha® Attract results fall with frequency compared with the BAHA/Ponto at frequencies above 2 kHz with a slope of about −10 dB/octave (Figure 10(a) and (b)). In the estimation of maximum power output, the Baha® Attract gave comparable levels to the Baha® connect system up to 1 kHz but fell with frequency at higher frequencies (van Barneveld et al., 2018). Compared with the simulations here, the high-frequency results were 5 to 10 dB lower, which could be related to differences in static force or skin thickness for the Baha® Attract system.

As a new designed skin-drive BCD, there are no clinical reports of the Adhear® BCD. But according to the comparison with the Baha®/Ponto in Figure 10(a) and (b), the responses obtained from the Adhear® were positive below 250 Hz but 10 to 30 dB lower than the Baha®/Ponto at frequencies between 250 and 4000 Hz. At frequencies above 4 kHz, the cochlear promontory vibration response with the Adhear® is similar to the Sophono® and the Baha® Attract BCDs. Moreover, the cochlear promontory vibrations with the Adhear® show the lowest levels of all BCDs at frequencies above 250 Hz, except for the Radioear B71 at frequencies above 4 kHz. The differences between the Adhear® and other BCDs are caused by the application method. The Adhear® is the only BCD which is attached to the skin with adhesive. The use of adhesive circumvents the need of a static force, but the static force enhances the transmission of BC sound applied at the skin surface (Khanna et al., 1976). Another reason for the less favorable results with the Adhear® is the small area that interfaces the device with the skin. A greater area has also been shown to be beneficial for BC transmission applied to the skin (Khanna et al., 1976).

The differences between the Baha®/Ponto and the other three direct-drive BCDs were small. Reinfeldt, Håkansson, Taghavi, Fredén Jansson, et al. (2015) reported clinical results of the first six patients with the BCI where the results with the BCI were better or similar compared with the results of a Ponto on a softband applied on the skin. However, such application of the Ponto is probably more similar to the Baha® Attract results than the Baha®/Ponto results here, as the Baha® Attract is also positioned on the skin at the same position. Figure 10(c) and (d) shows the total acceleration levels of the cochlea with 1 -V stimulation to the transducer to be around 5 dB greater for the BCI than for the Baha®/Ponto at frequencies above 0.5 kHz ipsilaterally and between 0.5 and 5 kHz contralaterally. These results are in line with cadaver head studies indicating that a stimulation position closer to the cochlea gives better cochlear vibration responses, especially at the ipsilateral side (Eeg-Olofsson et al., 2008; Håkansson, Eeg-Olofsson, Reinfeldt, Stenfelt, & Granström, 2008; Håkansson et al., 2010; Stenfelt & Goode, 2005b).

Huber et al. (2013) measured the cochlear promontory acceleration in five cadaver heads with the Bonebridge™ and the Baha® (BP 100). The results from that study implied that the Bonebridge™ gave up to 10 dB higher ipsilateral cochlear vibration and down to 5 dB worse contralateral cochlear vibration compared with the Baha®/Ponto. That result is also in line with the present simulation result where the Bonebridge™ gives up to 10 dB greater total acceleration compared with the Baha®/Ponto at the ipsilateral side (Figure 10(c)) and around 5 dB worse result at frequencies above 5 kHz at the contralateral side (Figure 10(d)). Moreover, the results of the Bonebridge™ are almost equal to the results of the BCI in Figure 10(c) and (d). Consequently, the differences in position, geometry, and interface between the two devices do not seem to influence the vibration of the cochlear promontory. In the study of maximum power output, the Bonebridge™ gave around 15 dB poorer output compared with the Baha® up to 2 kHz and similar levels at higher frequencies (van Barneveld et al., 2018). This fits well with the current simulations, as the Bonebridge™ (as well as the BCI) requires electromagnetic transmission over the intact skin which reduces the signal by around 10 dB (Håkansson et al., 2008). This means that the Bonebridge™ and BCI curves in Figure 10 should be downshifted by about 10 dB to account for the transcutaneous electromagnetic signal transmission.

There are some reports on the BC response with stimulation at the tooth (Gurgel & Shelton, 2013; Murray, Popelka, & Miller, 2011), but it is difficult to extract the transmission of vibrations from the tooth to the cochlea in those studies. Stenfelt and Håkansson (1999) compared hearing thresholds when the excitation was from the mastoid and the teeth. However, their measurements used the front teeth biting on a test rod, which is different from the SoundBite™. When the SoundBite™ was simulated with similar stimulation direction as the Baha®/Ponto, the total acceleration with 1 V to the transducer was similar for the two devices. However, different from the Bonebridge™ and the BCI, the cochlear acceleration levels with stimulation from the SoundBite™ were worse than from the Baha®/Ponto at frequencies between 0.5 and 5 kHz but better for the other frequencies. Consequently, the SoundBite™ produces lower cochlear promontory vibration responses in the mid-frequencies than the Baha®/Ponto but better at the low and high frequencies.

When the BCD simulations in this study are compared with other BCD evaluations, generally the results are comparable. However, some studies indicate superior performance of the BCDs, while other show inferior performance of the BCDs compared with the predictions here. One origin for such differences is differences in how the BCDs are evaluated. But some differences originate in differences in the transducer design and electronics of the BCDs. All BCDs except the Radioear B71 were evaluated with the same transducer model in the current simulations. This means that the comparison mainly evaluates the effects of the stimulation method (with a plate on the skin or rigidly attached in the skull) and position. The exact design of the BCDs transducers is proprietary knowledge and depending on design trade-offs; they can be better or worse than the predictions presented here. The transducer can also have a different resonance frequency, which means that it will provide greater stimulation levels at one frequency range and worse stimulation levels at a different frequency range compared with the modeled transducer in Figure 3. The driving voltage of the BCD is an additional important factor, for example, if only one or several batteries are used and if technology for increased voltage is used (e.g., step-up converter). Even if the specific design for each BCD is unknown, the simulations of the BCDs gave predictions based on cochlear vibrations that are in line with reports in the literature.

In summary, clinical comparisons between BCDs or experimental evaluations in cadaver heads show results that are in line with the findings in the current simulations. However, here, the BC transmission from all eight BCDs was evaluated for the first time in the same subject with a high-frequency resolution. This enables easy comparison of benefits and drawbacks in terms of BC sound transmission for the different positions and modes of application. As long as the application is to the skull bone, there are small differences in the BC transmission from the different positions to the ipsilateral cochlea, with a tendency of improved transmission the closer to the cochlea the stimulation position is (Figure 10(c)). When the stimulation is at the skin, the skin attenuates the vibration with up to 20 dB at higher frequencies, and the transmission can be significantly reduced if the stimulation area becomes small (Figure 10(a)).

## Conclusions

In this study, eight BCDs were simulated with the LiUHead model. When only the excitation related part of each BCDs was involved, the transmission properties of the BC sound were investigated with the same stimulation force. The vibration responses at the cochlear promontory of all BCDs are overall similar at frequencies below 500 Hz. At the high frequencies, above 4 kHz, the direct-drive BCDs show the greatest cochlear promontory vibration responses followed by the oral device. The skin-drive BCDs display the lowest cochlear promontory vibration response levels at the high frequencies.

When the effect of the transducer was incorporated in the simulations and the input signal was an equal voltage, all the direct-drive BCDs show similar cochlear promontory vibration responses where the BCI and Bonebridge™ were slightly better than the Baha®/Ponto and SoundBite™. The Sophono® and Baha® Attract, two skin-drive BCDs, gave similar cochlear promontory vibration responses as the Baha®/Ponto at frequencies up to 2 kHz but lower responses at high frequencies. The Radioear B71 showed the highest cochlear promontory vibration response levels at low frequencies but the lowest levels at the high frequencies. The Adhear®, however, presented the lowest cochlear promontory vibration responses of all BCDs at most of the frequencies. Although there are differences between the simulations and clinical evaluations of the BCDs, the results in this study provide insight to the function of the different types of BCDs and are helpful to understand the functions of the BCDs.
